# Toward Clinical Generative AI: Conceptual Framework

**DOI:** 10.2196/55957

**Published:** 2024-06-07

**Authors:** Nicola Luigi Bragazzi, Sergio Garbarino

**Affiliations:** 1 Human Nutrition Unit, Department of Food and Drugs University of Parma Parma Italy; 2 Department of Neuroscience, Rehabilitation, Ophthalmology, Genetics and Maternal/Child Sciences University of Genoa Genoa Italy

**Keywords:** clinical intelligence, artificial intelligence, iterative process, abduction, benchmarking, verification paradigms

## Abstract

Clinical decision-making is a crucial aspect of health care, involving the balanced integration of scientific evidence, clinical judgment, ethical considerations, and patient involvement. This process is dynamic and multifaceted, relying on clinicians’ knowledge, experience, and intuitive understanding to achieve optimal patient outcomes through informed, evidence-based choices. The advent of generative artificial intelligence (AI) presents a revolutionary opportunity in clinical decision-making. AI’s advanced data analysis and pattern recognition capabilities can significantly enhance the diagnosis and treatment of diseases, processing vast medical data to identify patterns, tailor treatments, predict disease progression, and aid in proactive patient management. However, the incorporation of AI into clinical decision-making raises concerns regarding the reliability and accuracy of AI-generated insights. To address these concerns, 11 “verification paradigms” are proposed in this paper, with each paradigm being a unique method to verify the evidence-based nature of AI in clinical decision-making. This paper also frames the concept of “clinically explainable, fair, and responsible, clinician-, expert-, and patient-in-the-loop AI.” This model focuses on ensuring AI’s comprehensibility, collaborative nature, and ethical grounding, advocating for AI to serve as an augmentative tool, with its decision-making processes being transparent and understandable to clinicians and patients. The integration of AI should enhance, not replace, the clinician’s judgment and should involve continuous learning and adaptation based on real-world outcomes and ethical and legal compliance. In conclusion, while generative AI holds immense promise in enhancing clinical decision-making, it is essential to ensure that it produces evidence-based, reliable, and impactful knowledge. Using the outlined paradigms and approaches can help the medical and patient communities harness AI’s potential while maintaining high patient care standards.

## Clinical Decision-Making and Clinical Intelligence

Clinical decision-making can be defined as a fundamental aspect of health care practice, encompassing a wide set of skills, competencies, processes, and outcomes through which clinicians gather and analyze relevant patient data; differentiate among various conditions; and diagnose, treat, and manage patient care, balancing the effectiveness, risks, and benefits of each treatment; patient preferences; and other related values within broader societal and cultural contexts and guidelines or standards of care [1-3].

Clinical decision-making involves a complex interplay of research and biomedical knowledge, experience, and intuitive understanding developed through years of practice, contextual analytical reasoning, patient-centeredness, and compliance with ethical standards and legal requirements, with the goal of arriving at optimal health outcomes for patients by making informed, evidence-based, and shared choices while ensuring patient autonomy and confidentiality [4,5].

The 4 major pillars of clinical decision-making are scientific evidence, clinical judgment (in some complex cases not isolated to 1 clinician but involving a team of health care professionals, each contributing their expertise), ethical considerations, and patient involvement, which are pivotal to the delivery of high-quality health care [6,7].

Clinical decision-making is not a static but rather a dynamic, multifaceted, iterative process based on reflective practice, which implies reviewing and auditing clinical decisions and outcomes to continuously learn and improve decision-making skills in the face of uncertainty and epistemic risks [5,8].

## The Advent of Generative Artificial Intelligence and Its Role in Supporting Clinical Decision-Making

Artificial intelligence (AI) [9] and, in particular, generative AI [10] have the potential to revolutionize the field of clinical decision-making with their advanced capabilities in data analysis and pattern recognition. However, together with their rise, there is a growing necessity to ensure that the knowledge used and produced is evidence based and reliable. This necessity stems from the potential risks and biases associated with AI-generated insights that may not align with established medical knowledge or practices.

Generative AI can process vast amounts of medical data, including patient records, imaging data, laboratory test results, other diagnostic inputs, and clinical studies, as well as research papers, to identify patterns and correlations that might be missed by clinicians. By analyzing patient data, generative AI can help in tailoring treatments to individual patients, improving the efficacy of therapies and reducing side effects, predicting disease progression and potential complications, aiding clinicians in proactive patient management, and assisting in diagnosing diseases, potentially identifying conditions earlier and more accurately than using traditional methods [11].

On the other hand, generative AI can produce “hallucinations” or even “fabrications” and “falsifications,” generating inaccurate or misleading information that does not accurately reflect the data it was trained on or reality [12,13], which is of particular concern in the medical realm.

Addressing these challenges requires a multifaceted approach, including improving data set quality and diversity, refining model architectures, and incorporating mechanisms for fact checking and validation. Moreover, developing methodologies for the model to express uncertainty or request clarification when generating outputs on topics in which it has less confidence could enhance reliability. In real-world clinical applications where accuracy and truthfulness are paramount, it is crucial to implement safeguards such as human oversight, rigorous testing across diverse scenarios, and continuous monitoring and updating of AI-based models to mitigate the risks associated with these inaccuracies.

In this conceptual paper, to address these concerns, we introduce 11 “verification paradigms,” with each paradigm being a unique method to verify the evidence-based nature of AI in clinical decision-making.

## Comparing Clinical Versus AI Reasoning

**Figure 1:**
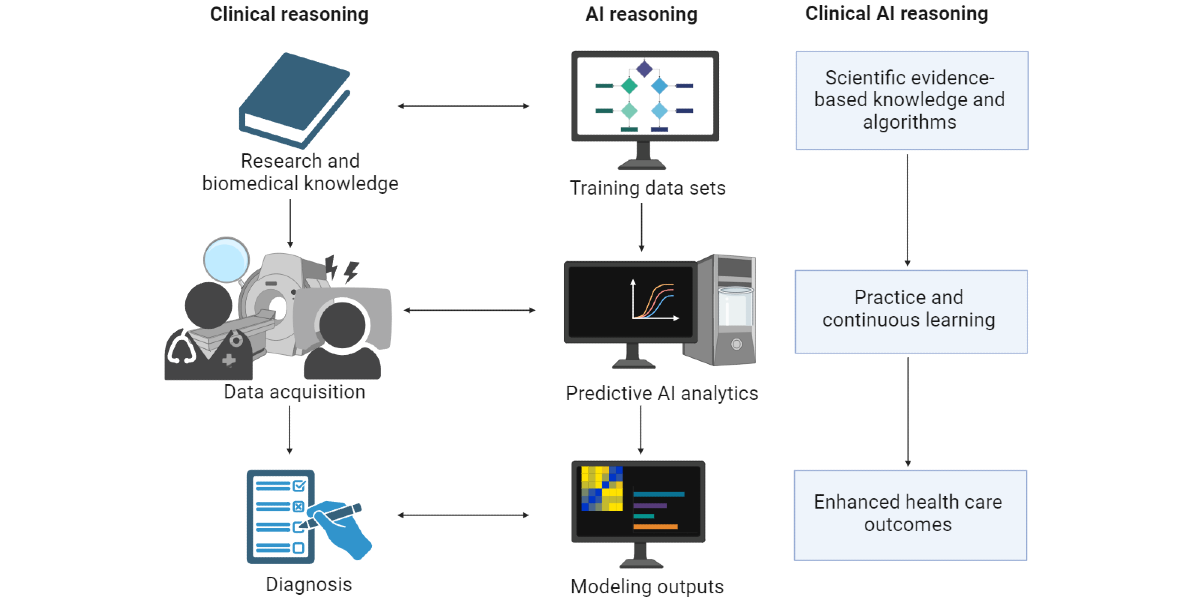
Integrating clinical expertise with artificial intelligence (AI) for enhanced health care outcomes—a schematic representation of the flow and interplay among traditional clinical reasoning, data acquisition, AI-driven predictive analytics, and the continuous learning cycle leading to improved patient care and diagnostics. This figure was created with BioRender.com.

Interesting parallelisms between clinical decision-making and AI reasoning can be drawn (Figure 1), especially in the context of frequentist and Bayesian thinking and large language models (LLMs) such as GPT-4, which use conditional probability, revealing an interesting interplay of similarities and contrasts [5].

In clinical decision-making, the reliance on scientific evidence mirrors AI’s dependence on extensive data sets for training. Clinicians, through years of practice, develop an intuitive sense of diagnosis and treatment. Clinical reasoning often involves abductive reasoning, which is a form of logical inference that starts with an observation or set of observations and then seeks to find the simplest and most likely explanation. In clinical practice, this means forming hypotheses based on symptoms and available data to diagnose a patient’s condition. AI, particularly in fields such as machine learning and diagnostic algorithms, also frequently uses abductive reasoning—AI-based systems are, indeed, designed to analyze data, identify patterns, and make predictions or decisions based on that analysis. In many ways, this mirrors the process of abductive reasoning in which the most likely conclusion is drawn from the available information. For example, in medical diagnostics, AI-based systems might analyze patients’ symptoms, medical history, and test results to suggest possible diagnoses. The aspect of human expertise underlying clinical reasoning somewhat parallels how AI-enhanced models develop a form of “intuition” from their vast training data [14,15].

When faced with complex cases, clinical decision-making often involves a collaborative approach among health care professionals, akin to the multifaceted approach of AI that integrates diverse data sources and algorithms. Ethical considerations and patient involvement are central to clinical decisions, much like how AI-based models need to be ethically aligned and user centric. Furthermore, both fields are dynamic and iterative—clinicians continually adapt their methods based on new research and patient feedback, similar to how AI-enhanced models evolve with new data and interactions.

On the AI side, traditional models often align with frequentist statistics, where the frequency of past events informs future predictions, somewhat like clinicians using epidemiological data. Modern AI, particularly in machine learning, uses Bayesian methods, updating the likelihood of outcomes with new data, reflecting how clinicians revise their hypotheses about diagnoses or treatments as new patient information comes to light. LLMs such as GPT-4 can predict outcomes based on conditional probability, which can be compared to clinicians using symptoms to predict diagnoses [16].

AI’s proficiency in pattern recognition and predictive analysis also finds a parallel in clinical practice, where patterns in patient symptoms and test results are crucial for effective decision-making. However, despite these parallelisms, significant differences remain, with AI lacking the empathetic and deeply intuitive component inherent in human decision-making and clinicians interpreting data within a broader human context, an ability that AI has yet to fully replicate.

In essence, while there are notable similarities in the use of statistical methods and data analysis between clinical decision-making and AI reasoning, the human aspects of intuition, empathy, and ethical considerations underscore the unique characteristics of each field. The future of health care may lie in the harmonious integration of these 2 domains, leveraging the strengths of each to enhance medical care and improve patient outcomes (Figure 1).

## Toward Clinical LLMs: Necessity of Verifying Evidence-Based Knowledge

However, the integration of generative AI into clinical decision-making necessitates a rigorous verification process to ensure the reliability and accuracy of the AI-generated insights. This verification is crucial because, as previously mentioned, AI-based models can sometimes generate conclusions based on flawed or biased data, leading to inaccurate or even harmful recommendations. It is essential that AI-generated advice aligns with current medical standards and best practices in addition to adhering to ethical standards, respecting patient autonomy, and ensuring equitable treatment [17,18].

Clinically oriented LLMs [19-25] such as ClinicalBERT, BlueBERT, CAML, DRG-LLaMA, GatorTronGPT, or PaLM have shown impressive capabilities, yet their application in clinical settings faces stringent requirements. Traditional methods of assessing these models’ clinical knowledge often depend on automated evaluations using narrow benchmarks. To overcome these shortcomings, Singhal et al [25] recently introduced MultiMedQA, a comprehensive benchmark that merges 6 medical question-answering data sets covering a range of areas from professional medicine to consumer queries and includes HealthSearchQA, a new data set of medically related web-based search questions. This novel approach includes a human evaluation framework that examines model answers across various dimensions, namely, accuracy, understanding, reasoning, potential harm, and bias. The authors tested both PaLM and its instruction-tuned version, Flan-PaLM, on MultiMedQA. Flan-PaLM, using diverse prompting techniques, set a new standard in accuracy across all MultiMedQA multiple-choice data sets, including MedQA, MedMCQA, PubMedQA, and MMLU clinical topics, achieving a remarkable 67.6% accuracy in MedQA (US Medical Licensing Examination–style questions), which is >17% higher than the previous best. However, human assessments uncovered significant shortcomings. To address these, the authors introduced “instruction prompt tuning,” an efficient method for adapting LLMs to new domains with just a few examples. The resultant model, Med-PaLM, shows promise, yet it still does not match clinician performance even though the authors could observe that model scale and instruction prompt tuning significantly enhance comprehension, knowledge recall, and reasoning.

A further risk is that LLMs might reinforce existing biases and provide inaccurate medical diagnoses, potentially leading to detrimental effects on health care. Zack et al [26] aimed to evaluate whether GPT-4 harbors biases that could influence its application in health care settings. Using the Azure OpenAI interface, the authors scrutinized GPT-4 for racial and gender biases and assessed the impact of such biases on four clinical applications of LLMs—(1) medical education, (2) diagnostic reasoning, (3) development and implementation of clinical plans, and (4) subjective patient evaluations—involving experiments using prompts mimicking typical GPT-4 use in clinical and medical educational settings and drawing from *New England Journal of Medicine* Healer clinical vignettes and research on implicit bias in health care. The study compared GPT-4’s estimates of demographic distributions of medical conditions against actual US prevalence data. For differential diagnosis and treatment planning, the research analyzed variations across demographic groups using standard statistical methods to identify significant differences. The study revealed that GPT-4 inadequately represents demographic diversity in medical conditions, often resorting to stereotypical demographic portrayals in clinical vignettes. The differential diagnoses generated by GPT-4 for standardized clinical vignettes tended to reflect biases associated with race, ethnicity, and gender. Furthermore, the model’s assessments and plans demonstrated a notable correlation between demographic characteristics and recommendations for costlier procedures, as well as varied perceptions of patients.

All this, taken together, suggests the potential role of LLMs in medicine, but human evaluations also highlight the current models’ limitations, underscoring the importance of comprehensive evaluation frameworks and continued methodological advancements to develop safe, effective LLMs for clinical use.

## Implementing “Verification Paradigms”: A Comprehensive Evaluation Framework

### Overview

Several “simulation and scenario testing” or “verification” paradigms can be particularly effective in verifying the evidence-based nature of generative AI in clinical decision-making. The 11 paradigms proposed in this paper were devised following thorough familiarization with existing literature and extensive consultation with experts in the field to ensure that the methodologies were not only grounded in the latest academic research and theoretical frameworks but also shaped by practical insights and recommendations from medical professionals and AI technology specialists (Textbox 1 and Table 1).

**Overview of the verification paradigms.**

**Verification paradigms and brief description**

- Quiz, vignette and knowledge survey: uses clinical scenarios to test artificial intelligence (AI)’s medical knowledge and reasoning.
- Historical data comparison: compares AI recommendations with known clinical outcomes to gauge accuracy.
- Expert consensus: evaluates AI-generated diagnoses or treatment plans against expert medical opinion.
- Cross-discipline validation: verifies AI insights with professionals from various medical disciplines for comprehensive evaluation.
- Rare or complex simulation and scenario testing: assesses AI’s ability to handle rare and complex medical cases through simulated scenarios.
- False myth: tests AI’s capability to identify and reject medical myths or outdated concepts.
- Challenging (or controversial) question: presents AI with complex medical questions to evaluate its nuanced understanding and reasoning.
- Real-time monitoring: monitors AI recommendations in clinical settings to observe real-world efficacy and safety.
- Algorithm transparency and audit: focuses on the transparency of AI’s decision-making process and its ability to be audited.
- Feedback loop: involves continuous AI improvement based on feedback from practical applications and outcomes.
- Ethical and legal review: regularly reviews AI recommendations to ensure that they adhere to ethical guidelines and legal standards.

**Table 1:** Verification paradigms with their strengths and weaknesses.

Verification paradigm	Strengths	Weaknesses
Quiz, vignette, and knowledge survey	Comprehensive evaluation Real-world relevance Assessment of contextual understanding and probabilistic reasoning	Complex to design Resource intensive Potential bias in test creation
Historical data comparison	Real-world applicability Evidence-based evaluation Objective benchmarking	Dependent on data quality Historical bias May not capture AI’s^a^ potential for novel insights
Expert consensus	Leverages human expertise Valuable in complex cases Incorporates ethical judgment	Subjective Time-consuming Potential for expert bias
Cross-discipline validation	Comprehensive evaluation from multiple perspectives Mitigates the risk of siloed decision-making	Coordination challenges Requires broad expert availability
Rare or complex simulation and scenario testing	Reveals AI’s capabilities in handling diversity Can identify areas for innovation	Potentially limited by available data Resource intensive
False myth	Tests AI’s current knowledge base Assesses ability to discern evidence-based information	Requires careful selection of myths Risk of reinforcing incorrect information
Challenging (or controversial) question	Evaluates AI’s handling of ambiguity and complexity Assesses balance of different viewpoints	Subjective evaluation criteria Depends on quality of input questions
Real-time monitoring	Direct insight into practical impact Simulates real-world testing	Requires controlled clinical environment Ethical concerns with experimental use
Algorithm transparency and audit	Enhances trust and understanding Facilitates regulatory compliance	Complexity for end users Risk of exposing proprietary information
Feedback loop	Ensures continuous improvement Adapts to changing medical knowledge	Requires ongoing effort and resources Dependence on quality of feedback
Ethical and legal review	Safeguards patient rights Ensures adherence to ethical guidelines	Time-consuming Needs multidisciplinary expertise

^a^AI: artificial intelligence.

### The Quiz, Vignette, and Knowledge Survey Paradigm

This approach involves assessing the AI’s proficiency in various domains, such as medical knowledge and diagnostic reasoning, and its understanding of therapeutic interventions by using quizzes, vignettes, and validated knowledge surveys designed to mimic real-world clinical scenarios [27]. This would require the AI to have not only a vast knowledge base of medical information but also, and especially, the ability to apply this knowledge contextually, thus demonstrating an understanding of the nuances of patient presentations and how they correlate with various medical conditions and treatments. In addition, this format could incorporate elements of both frequentist and Bayesian thinking, reflecting the probabilistic nature of clinical reasoning—in other words, as previously mentioned, the AI would have to weigh the likelihood of different diagnoses based on the presented symptoms and history, similar to how clinicians use Bayesian reasoning to update their probability assessments as new information becomes available.

This approach has a number of strengths, including comprehensive evaluation, real-world relevance, contextual understanding, probabilistic reasoning assessment, and adaptability to new information. On the other hand, it suffers from some weaknesses, such as design complexity and resource intensiveness, potential bias in test creation, and lack of interdisciplinary evaluation.

Currently, this approach is the most leveraged. An extensive body of literature has found that LLMs such as ChatGPT can successfully pass medical examinations [28] although with varying degrees of heterogeneity and variability [29], exhibiting strong abilities in explanation, reasoning, memory, and accuracy. On the other hand, LLMs struggle with image-based questions [30] and, in some circumstances, lack insight and critical thinking skills [31].

Some of the studies that have exploited quizzes, vignettes, and validated knowledge surveys [32,33] have quantified the fluency and accuracy of AI-based tools using validated and reliable instruments such as the “Artificial Intelligence Performance Instrument” [32]. This tool includes 9 items related to medical and surgical history, namely, symptoms, physical examination, diagnosis, additional examinations, management plan, and treatments. The Artificial Intelligence Performance Instrument score ranges from 0 (“inadequate clinical case management by the AI”) to 20 (“excellent clinical case management by the AI”). This score can be further subdivided into 4 subscores: patient feature, diagnosis, additional examination, and treatment score.

### The Historical Data Comparison Paradigm

This approach involves comparing AI-generated recommendations with outcomes from historical data—by analyzing cases in which the clinical outcomes are well known, one can assess how well the AI’s suggestions would have aligned with actual scenarios. This would help in the comprehension of the AI’s accuracy in real-world health care settings, providing insights into its potential benefits and limitations. This is a crucial step in understanding AI’s performance and guiding its integration into clinical practice, ensuring that AI-supported decisions are in line with evidence-based medical standards and, ultimately, enhance patient care outcomes.

Strengths of this approach include real-world applicability, evidence-based evaluation, and objective benchmarking by offering a clear, objective, data-driven, and evidence-based way to benchmark AI performance against known outcomes, facilitating a straightforward and comprehensive assessment of its accuracy. Furthermore, this method enables the identification of potential gaps and improvement areas—through direct comparison with historical outcomes, specific areas in which AI recommendations may fall short can be identified, guiding further refinements. Demonstrating AI’s ability to match or surpass historical outcomes can build trust among clinicians and patients regarding AI’s utility in health care. However, this method has some weaknesses, too, including dependence on data quality in that the approach is heavily reliant on the availability and quality of historical data, with poor data quality skewing results and misleading about AI’s true performance. In addition, historical data may contain biases (eg, diagnostic, treatment, or outcome biases), which can inadvertently be reinforced by AI, affecting the fairness and accuracy of its recommendations. This shortcoming is known as “historical bias,” which arises when the data or *corpora* used to train AI-based tools no longer accurately reflect the current reality. The potential lack of novel insights is another limitation as this method benchmarks against known outcomes and may not fully capture AI’s potential to provide novel insights or diagnose conditions that were previously undetected or misdiagnosed. Furthermore, this paradigm evaluates AI against past standards of care, which may not account for advancements in medical knowledge or changes in clinical guidelines over time (“static evaluation”), and its performance on complex, multifactorial cases might not be accurately assessed if historical data are limited or if such cases were managed differently due to evolving standards of care.

Currently, to the best of our knowledge, no published studies have leveraged this approach in the biomedical arena.

### The Expert Consensus Paradigm

In this paradigm, AI-generated diagnoses or treatment plans are evaluated by a panel of medical experts, with the consensus among these experts on the validity of the AI’s recommendations serving as a measure of their reliability. This paradigm is particularly useful in assessing the AI’s performance in complex cases in which human expertise is invaluable, ranging from the psychiatric field in dealing with issues such as suicide risk assessment [34] to occupational medicine [35]; oncology, with the management of malignancies [36]; and complex surgical procedures such as bariatric surgery [37].

Strengths include high-quality validation of AI’s performance, ensuring that AI-generated recommendations are thoroughly vetted by experts, and bringing a high level of scrutiny and quality control that is particularly important in complex medical fields. Incorporation of human expertise and adaptability to complex cases are other strengths by relying on medical experts to evaluate AI advice and integrating nuanced human judgment and clinical experience that AI might lack or in those instances for which AI algorithms might not have sufficient training data or might lack the capability to understand context deeply. Furthermore, expert feedback provides continuous learning opportunities, offering a platform for AI-based systems to be continuously updated and improved, enhancing their accuracy and reliability over time. This leads to heightened acceptance of AI tools as having a consensus from medical experts can increase trust among health care providers and patients in AI-generated diagnoses or treatment plans.

On the other hand, expert feedback is time and resource intensive—gathering a panel of experts and reaching a consensus can be time-consuming and expensive, which may not be feasible for every clinical decision or in settings with limited resources. In addition, despite being experts, humans are subject to biases that might affect their judgment, potentially leading to the validation of inaccurate AI recommendations. Scalability issues represent a further shortcoming—the approach may not scale well to everyday clinical practice, where quick decision-making is often required and the luxury of convening an expert panel for each AI recommendation is not practical. Furthermore, variability in expert opinion could lead to inconsistent validation of AI-generated recommendations and uncertainty in their reliability. Finally, there is a risk that this paradigm could discourage direct validation of AI algorithms through objective measures or independent verification, potentially overlooking errors or biases in the AI-based systems themselves.

### The Cross-Discipline Validation Paradigm

This paradigm is rooted in the understanding that health care delivery increasingly relies on the expertise and coordination of diverse professionals to address complex health issues effectively. This approach recognizes that no single professional has all the knowledge and skills necessary to provide comprehensive care, especially in cases that involve multifaceted medical, psychological, social, and ethical considerations. As clinical decision-making is seen as a multidisciplinary teamwork process, this verification paradigm involves cross-verifying AI-generated insights with experts from various medical disciplines. For example, a diagnosis made by an AI based on radiology images could be evaluated by experts in radiology, oncology, and pathology. This multidisciplinary approach ensures comprehensive evaluation and mitigates the risk of siloed decision-making, which is known to result in incomplete information, lack of coordination, and duplication of efforts, leading to inefficient care, higher costs, increased risk of medical errors, and decreased patient satisfaction, ultimately impacting the quality of patient care and health outcomes.

Currently, little is known about the multidisciplinary nature of LLMs. Li et al [38] evaluated the proficiency of AI-based tools in addressing interdisciplinary queries in cardio-oncology, leveraging a questionnaire consisting of 25 questions compiled based on the 2022 European Society of Cardiology guideline on cardio‐oncology. ChatGPT-4 showed the highest percentage of good responses at 68%, followed by Bard, Claude 2, and ChatGPT-3.5 at 52% and LLaMA 2 at 48%. A specific area of concern was in treatment and prevention, where all LLMs scored poorly or borderline, particularly when their advice deviated from current guidelines, such as the recommendation to interrupt cancer treatment for patients with acute coronary syndrome. Other studies have assessed LLMs as support tools for multidisciplinary tumor boards in the planning of therapeutic programs for patients with cancer [39,40].

### The Rare or Complex Simulation and Scenario Testing Paradigm

In this method, the AI-based tool is tested against a variety of simulated clinical scenarios, including rare and complex cases such as frail patients with multiple comorbidities, unusual presentations of diseases, or cases in which symptoms are ambiguous or misleading. This comprehensive testing can identify areas for innovation and reveal the strengths and limitations of the AI-based tool in diverse clinical situations, such as AI’s capabilities in handling diversity. Conversely, this paradigm can be resource intensive and potentially limited by available data.

A recent study [41] explored ChatGPT’s potential contributions to the diagnosis and management of rare and complex diseases, such as idiopathic pulmonary arterial hypertension, Klippel-Trenaunay syndrome, early-onset Parkinson disease, and Rett syndrome. LLMs can detect the disease early through AI-driven analysis of patient symptoms and medical imaging data, rapidly analyze an extensive body of biomedical literature for a better understanding of the mechanisms underlying the disease, and offer access to the latest research findings and personalized treatment plans.

Another study [42] examined the efficacy of 3 popular LLMs in medical education, particularly for diagnosing rare and complex diseases, and explored the impact of prompt engineering on their performance. Experiments were conducted on 30 cases from a diagnostic case challenge collection using various prompt strategies and a majority voting approach to compare the LLMs’ performance against human consensus and MedAlpaca, an LLM designed for medical tasks. The findings revealed that all tested LLMs surpassed the average human consensus and MedAlpaca’s performance by margins of at least 5% and 13%, respectively. In categories of frequently misdiagnosed cases, Google Bard equaled MedAlpaca but exceeded human consensus by 14%. GPT-4 and GPT-3.5 showed superior performance over MedAlpaca and human respondents in often moderately misdiagnosed cases, with minimum accuracy improvements of 28% and 11%, respectively. Using a majority voting strategy, particularly with GPT-4, yielded the highest overall accuracy across the diagnostic complex case collection. On the Medical Information Mart for Intensive Care III data sets, Google Bard and GPT-4 reached the highest diagnostic accuracy scores of 93% with multiple-choice prompts, whereas GPT-3.5 and MedAlpaca scored 73% and 47%, respectively.

### The False Myth Paradigm

This paradigm involves deliberately introducing known medical myths or outdated concepts into the AI’s training data. The AI’s ability to identify and reject these myths serves as a test of its understanding of current medical knowledge and its ability to discern evidence-based information. On the other hand, this approach requires a careful selection of myths and, if used in an inappropriate way, can reinforce incorrect information.

A few studies have harnessed this approach [43,44]. These studies evaluated the accuracy of 2 AI tools, ChatGPT-4 and Google Bard, in debunking 20 sleep-related myths using a 5-point Likert scale for falseness and public health significance and compared their performance with expert opinions. ChatGPT labeled 85% of the statements as either “false” (45%) or “generally false” (40%), showing high reliability in identifying inaccuracies, especially regarding sleep myths surrounding timing, duration, and behaviors during sleep. The tool demonstrated varying success in other categories such as presleep behaviors and brain function related to sleep. On a 5-point Likert scale, ChatGPT scored an average of 3.45 (SD 0.87) in identifying the falseness of statements and 3.15 (SD 0.99) in understanding their public health significance, indicating a good level of accuracy and understanding. Similarly, Google Bard identified 19 out of 20 statements as false, which was not significantly different from ChatGPT-4’s accuracy. Google Bard’s average falseness rating was 4.25 (SD 0.70), with skewness of −0.42 and kurtosis of −0.83, indicating a distribution with fewer extreme values compared to that of ChatGPT-4. For public health significance, Google Bard scored an average of 2.4 (SD 0.80), with skewness and kurtosis of 0.36 and −0.07, respectively, suggesting a more normal distribution than that of ChatGPT-4. The intraclass correlation coefficient between Google Bard and sleep experts was 0.58 for falseness and 0.69 for public health significance, showing moderate agreement. Text mining analysis showed that Google Bard focused on practical advice, whereas ChatGPT-4 emphasized theoretical aspects. A readability analysis found that Google Bard’s responses matched an 8th-grade reading level, making them more accessible than ChatGPT-4’s, which aligned with a 12th-grade level.

### The Challenging (or Controversial) Question Paradigm

In this paradigm, the AI-based tool is presented with controversial or complex medical questions that do not have straightforward answers. The way in which AI navigates these questions, balancing different viewpoints and evidence, can reveal its depth of understanding and its ability to handle nuanced medical issues. In the realm of medicine, evidence is hierarchical, with systematic reviews and meta-analyses at the top. An analytical evaluation would consider how the AI prioritizes, evaluates, and appraises different levels of evidence and whether it can differentiate between high-quality and lower-quality studies. In addition, AI should detect and minimize biases present in medical literature and data sources. Analytically, this involves evaluating the algorithms for their ability to identify potential biases in studies (eg, publication bias and selection bias) and adjust their conclusions accordingly. Shortcomings of this paradigm include subjective evaluation criteria and dependence on the quality of input questions.

A few studies [45,46] have assessed the skills of AI-based tools in understanding or generating complex and nuanced clinical documents, such as guidelines.

### The Real-Time Monitoring Paradigm

In this paradigm, the AI’s recommendations are implemented in a controlled clinical environment, and patient outcomes are closely monitored, simulating a randomized controlled trial (RCT). This real-world testing provides valuable feedback on the AI’s efficacy and safety in actual clinical settings.

While this paradigm can provide direct insights into practical impact and simulate real-world testing, it requires a controlled clinical environment and may be limited by ethical concerns related to the experimental use of AI.

So far, only a few RCTs have been implemented. A recent blinded RCT [47] explored the efficacy of ChatGPT alongside traditional typing and dictation methods in assisting health care providers with clinical documentation, specifically in writing a history of present illness based on standardized patient histories. A total of 11 participants, including medical students, orthopedic surgery residents, and attending surgeons, were tasked with documenting history of present illness using 1 of the 3 methods for each of the 3 standardized patient histories. The methods were assessed for speed, length, and quality of documentation. Results indicated that, while dictation was the fastest method and resulted in longer and higher-quality patient histories according to the Physician Documentation Quality Instrument score, ChatGPT ranked intermediate in terms of speed. However, ChatGPT-generated documents were more comprehensive and organized than those produced through typing or dictation. A significant drawback noted was the inclusion of erroneous information in slightly more than one-third of ChatGPT-generated documents, raising concerns about accuracy. In addition, there was a lack of consensus among reviewers regarding the quality of patient histories.

In another controlled trial [48], ChatGPT’s utility in providing empathetic responses to people with multiple sclerosis was assessed. The study recruited a sample of 1133 participants (mean age 45.26, SD 11.50 years; 68.49% female), who were surveyed through a web-based form distributed via digital communication platforms. Participants, blinded to the authors of the responses, evaluated alternate responses to 4 questions on a Likert scale from 1 to 5 for overall satisfaction and used the Consultation and Relational Empathy scale for assessing perceived empathy. Results showed that ChatGPT’s responses were perceived as significantly more empathetic than those from neurologists. However, there was no significant association between ChatGPT’s responses and mean satisfaction. College graduates were significantly less likely to prefer ChatGPT’s responses compared to those with a high school education.

### The Algorithm Transparency and Audit Paradigm

This paradigm focuses on the transparency of the AI algorithms and the ability to audit their decision-making processes. By understanding how the AI-based tool arrives at its conclusions, clinicians can better assess the validity of its recommendations, which is crucial for building trust in AI-based systems among health care professionals.

Strengths include improved decision-making and enhanced trust and confidence by demystifying how decisions are made, thus building trust among clinicians and patients, crucial for the acceptance and integration of AI in health care. Clinicians can make more informed decisions by understanding the reasoning behind AI recommendations, potentially leading to better patient outcomes. AI-based tools can also facilitate regulatory compliance—transparency is key to meeting regulatory standards for medical devices and software, including AI-based systems used in health care. AI enables continuous improvement as a transparent decision-making process allows for easier identification of errors or biases in the AI system, facilitating ongoing refinement and improvement. Furthermore, exposing the decision-making process has educational benefits for health care professionals, helping them understand complex AI methodologies and enhancing their ability to work alongside AI tools. On the other hand, this approach has some weaknesses that should be acknowledged, including complexity for end users—AI decision-making processes, especially in deep learning, can be incredibly complex and difficult for end users to understand, potentially limiting the effectiveness of transparency. Understanding and trusting the AI process might lead some clinicians to overrely on AI recommendations without applying their judgment, especially in ambiguous or complex cases. Complete transparency might expose proprietary algorithms to potential theft or misuse, challenging companies to balance transparency with protecting their intellectual property. Moreover, there is potential room for misinterpretation—there is a risk that transparency could lead to misinterpretation of how AI algorithms work, especially without a strong foundation in data science or AI methodologies among health care professionals. Finally, developing transparent AI systems that are also understandable to clinicians requires significant resources, including time and expertise, potentially slowing down innovation.

### The Feedback Loop Paradigm

This approach involves the continuous updating of the AI system based on feedback from its practical applications, with clinicians providing feedback on the AI’s performance, which is then used to refine and improve the AI models. This iterative, ongoing process ensures that the AI-based system properly evolves and adapts to changing medical knowledge and practices. Conversely, it also requires ongoing efforts and resources in addition to depending on the quality of the feedback.

A few studies have investigated reproducibility and repeatability [49,50]. In a study [49] involving emergency physicians, 6 unique prompts were used in conjunction with 61 patient vignettes to assess ChatGPT’s ability to assign Canadian Triage and Acuity Scale scores through 10,980 simulated triages. ChatGPT returned a Canadian Triage and Acuity Scale score in 99.6% of the queries. In terms of temporal reproducibility and repeatability, the study found considerable variation in the results—21% due to repeatability (using the same prompt multiple times) and 4% due to reproducibility (using different prompts). ChatGPT’s overall accuracy in triaging patients was 47.5%, with an undertriage rate of 13.7% and an overtriage rate of 38.7%. Of note, providing more detailed prompts resulted in slightly greater reproducibility but did not significantly improve accuracy.

In another study [50] assessing ChatGPT’s proficiency in answering frequently asked questions about endometriosis, detailed internet searches were used to compile questions, which were then aligned with the European Society of Human Reproduction and Embryology (ESHRE) guidelines. An experienced gynecologist rated ChatGPT’s responses on a scale from 1 to 4. To test repeatability, each question was asked twice, with reproducibility determined by the consistency of ChatGPT’s scoring within the same category for repeated questions. Of the frequently asked questions, 91.4% (n=71) were answered completely, accurately, and sufficiently by ChatGPT. The model showed the highest accuracy in addressing symptoms and diagnosis (16/17, 94% of the questions) and the lowest accuracy in treatment-related questions (13/16, 81% of the questions). Among the 40 questions related to the ESHRE guidelines, 27 (68%) were rated as grade 1, a total of 7 (18%) were rated as grade 2, and 6 (15%) were rated as grade 3. The reproducibility rate was highest (100%) for questions in the categories of prevention, symptoms and diagnosis, and complications. However, it was lowest for questions aligned with the ESHRE guidelines, at 70%.

These contrasting findings warrant further investigation.

### The Ethical and Legal Review Paradigm

The “ethical and legal review paradigm” emphasizes the importance of ensuring that AI recommendations in health care settings adhere to established ethical guidelines and legal standards, which involves regular review rounds of the AI’s recommendations by an ethics committee or legal team. This is particularly important in sensitive areas such as critical care, emergency management, end-of-life care, or genetic testing, where the stakes of decisions are particularly high and the moral and legal implications are significant. This approach aims to safeguard patients’ rights, maintain trust in AI-assisted health care, and ensure that the implementation of AI technologies in medicine is both ethically sound and legally compliant [51,52].

The deployment of AI-based tools such as ChatGPT in sensitive fields raises, indeed, several ethical and legal concerns. One significant issue is the potential for bias in AI algorithms, which can lead to unfair or incorrect outcomes. Moreover, the use of AI in these fields touches on privacy concerns, especially with the processing of personal data. Furthermore, issues regarding accountability and liability for malpractices and bad outcomes associated with AI-influenced LLM medical decision-making represent an emerging topic in the arena of legal medicine and, more broadly, forensic science.

These concerns underscore the need for strict ethical guidelines and robust legal frameworks governing AI use in biomedical and clinical practices, with the final goal of leveraging AI’s strengths while mitigating its limitations, ensuring that it serves as a tool for progress rather than a source of bias and error [52,53].

## Integrating the “Verification Paradigms”

**Figure 2:**
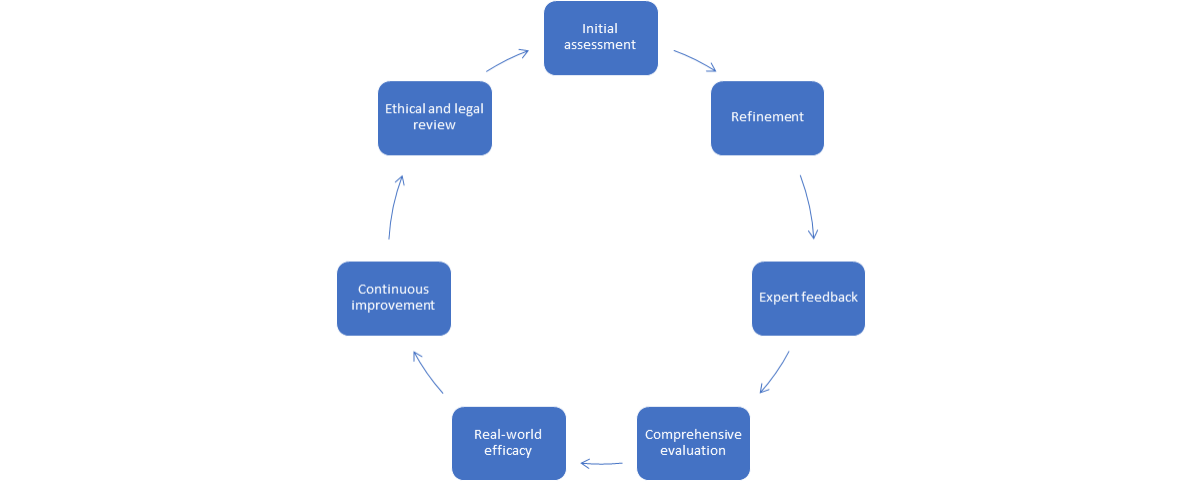
Integrating verification paradigms for artificial intelligence in health care.

These various paradigms for assessing AI in health care contexts underscore the multifaceted and complex nature of integrating AI technologies such as ChatGPT into medical practices. These paradigms reflect a concerted effort to evaluate AI systems’ proficiency, ethical alignment, and practical utility in clinical settings comprehensively. Each of these paradigms offers a unique perspective and method for verifying the reliability and accuracy of generative AI in clinical decision-making, and they can be used in combination to provide a robust validation framework (Tables 2 and 3 and Figure 2).

It is of paramount importance to note that all these paradigms do not necessarily have the same weight or importance; their relevance can vary depending on the context, the specific health care domain, and the goals of the AI system being assessed. Integrating and combining these paradigms can provide a comprehensive, robust evaluation framework that leverages the strengths of each approach while mitigating their individual limitations.

Contextual or clinical relevance can be used to prioritize these approaches—in clinical settings in which decision-making is complex and highly nuanced (eg, oncology or psychiatry), paradigms that emphasize expert consensus and cross-discipline validation may be more critical, whereas for emerging treatments or rare diseases, paradigms focusing on simulation and scenario testing and challenging questions can be invaluable to explore AI’s capacity to contribute novel insights or support rare condition management. In contexts in which AI is being directly implemented into clinical workflows and related follow-up, real-time monitoring and feedback loop paradigms become essential to ensure patient safety and system efficacy.

Combining paradigms for comprehensive evaluation requires a “layered, sequential, strategic integrative approach,” starting with broad assessments such as the quiz, vignette, and knowledge survey paradigm to gauge general knowledge and reasoning abilities, followed by more specific tests such as historical data comparison for accuracy in real-world scenarios and expert consensus for nuanced judgment calls. The cross-discipline validation paradigm can be harnessed to assess AI’s recommendations from multiple professional perspectives, ensuring a holistic evaluation of AI’s clinical recommendations. Throughout all stages of evaluation, the ethical and legal review paradigm is continuously applied to ensure adherence to ethical standards and legal requirements, safeguarding patient rights and data privacy.

**Table 2:** Overview of the layered integrative approach for evaluating artificial intelligence (AI) in health care, delineating the structured, multistage framework for the comprehensive assessment and continuous improvement of AI systems.

Stage	Verification paradigm	Objective	Integration
Initial assessment	Quiz, vignette, and knowledge survey	To gauge the AI’s foundational medical knowledge and its ability to apply this knowledge in simulated real-world scenarios	Forms the baseline assessment of the AI’s capabilities, setting the stage for more targeted evaluations
Refinement	Historical data comparison	To refine the AI’s understanding and application of medical knowledge by comparing its recommendations or diagnoses against known outcomes from historical data	Uses the insights gained from initial assessments to focus on areas requiring improvement, ensuring that the AI’s recommendations are grounded in real-world evidence
Expert feedback	Expert consensus	To incorporate nuanced clinical insights and expert judgments into the AI’s learning, ensuring that it aligns with current clinical practices and expert opinions	Builds on the refined knowledge base by integrating expert clinical insights, further improving the AI’s decision-making processes
Comprehensive evaluation	Cross-discipline validation	To evaluate the AI’s recommendations and diagnostics across various medical disciplines, ensuring a comprehensive and holistic assessment	Leverages the foundational knowledge, refined understanding, and expert insights to test the AI’s capabilities in a multidisciplinary context, identifying any gaps or biases
Complexity handling	Rare or complex simulation and scenario testing	To test the AI’s ability to handle complex, rare, or novel medical scenarios, ensuring that it can adapt to a wide range of clinical challenges	Uses the comprehensive evaluations as a foundation to challenge the AI with scenarios that require sophisticated reasoning, further refining its decision-making abilities
Knowledge accuracy	False myth	To ensure that the AI’s current knowledge base is accurate and up-to-date, identifying and correcting any misconceptions or outdated information	Builds on the previous layers by specifically targeting and rectifying inaccuracies in the AI’s knowledge, ensuring reliability
Complexity and nuance handling	Challenging (or controversial) question	To evaluate the AI’s ability to navigate complex medical questions that may not have straightforward answers, assessing its reasoning in ambiguous situations	Further refines the AI’s decision-making process by exposing it to nuanced clinical scenarios, enhancing its ability to provide balanced and informed recommendations
Real-world efficacy	Real-time monitoring	To monitor the AI’s recommendations and diagnoses in real-world clinical settings, assessing its practical efficacy and safety	Applies all previous layers of assessment in a live clinical environment, providing direct feedback on the AI’s performance and areas for improvement
Transparency and trust	Algorithm transparency and audit	To ensure that the decision-making processes of the AI are transparent and understandable, building trust among health care providers and patients	Uses insights from real-world applications and previous evaluations to demystify the AI’s logic, ensuring that it is both effective and comprehensible
Continuous improvement	Feedback loop	To continuously refine and improve the AI system based on real-world data, feedback, and evolving medical knowledge	Represents the culmination of the integrative approach, in which feedback from all previous stages is used to iteratively enhance the AI system, ensuring that it remains effective, safe, and ethically compliant over time
Ethical and legal compliance	Ethical and legal review	To ensure that all AI recommendations and processes adhere to established ethical guidelines and legal standards	Runs parallel to all stages, providing a constant check on the AI’s compliance with ethical norms and legal requirements, safeguarding against potential malpractices, and ensuring that patient rights are protected

**Table 3:** Engagement and impact of key health care stakeholders—physicians, patients, nurses, administrators, artificial intelligence (AI) developers, ethicists, and regulators—across various AI evaluation paradigms, highlighting their roles and interactions in the process of assessing and integrating AI technologies in health care.

Verification paradigm	Stakeholders						
	Physicians	Patients	Nurses	Health care administrators	AI developers	Ethicists	Regulators
Quiz, vignette, and knowledge survey	Participate in creating and testing	May be participants in scenarios	Assist in scenario design	Oversee implementation	Design relevant quizzes and surveys	Evaluate scenario ethics	Establish standards for testing
Historical data comparison	Use outcomes to validate AI	Benefit from improved outcomes	Observe AI’s real-world accuracy	Use data for strategic decisions	Analyze comparison outcomes for improvement	Assess the ethical use of historical data	Monitor data use and outcomes
Expert consensus	Contribute expertise	Trust in consensus-driven AI	Support expert consensus	Involved in consensus building	Incorporate expert feedback	Participate in consensus discussions	Ensure that expert consensus meets guidelines
Cross-discipline validation	Collaborate across specialties	Benefit from holistic care approaches	Facilitate multidisciplinary care	Ensure interdisciplinary cooperation	Work with diverse health care teams	Ensure ethical cross-discipline validation	Regulate multidisciplinary validation processes
Rare or complex simulation and scenario testing	Engage in scenario creation and testing	Receive personalized care for rare conditions	Involved in patient care scenarios	Plan for innovative care solutions	Design simulations for complex conditions	Scrutinize simulations for ethical considerations	Oversee testing for safety and efficacy
False myth	Input on relevant myths	Protected from misinformation	Educate patients on myths vs facts	Promote accurate patient education	Correct and update AI knowledge	Highlight the ethical handling of myths	Regulate misinformation management
Challenging (or controversial) question	Address complex questions	Empowered by nuanced AI assistance	Assist in managing complex cases	Address policy implications	Develop algorithms for nuanced questions	Engage in ethical debates	Set standards for addressing controversial topics
Real-time monitoring	Monitor patient outcomes	Directly affected by AI recommendations	Monitor and report on patient responses	Supervise operational integration	Refine AI through real-time data	Monitor ethical implications of real-time use	Ensure patient safety in real-time monitoring
Algorithm transparency and audit	Require understanding of AI decisions	Seek transparency for trust	Advocate for clear AI explanations	Demand system transparency	Ensure algorithmic transparency	Advocate for transparent decision-making	Enforce transparency and auditability
Feedback loop	Provide clinical feedback	Benefit from ongoing improvements	Offer practical feedback	Implement system feedback	Use feedback for technical refinement	Provide ethical oversight in feedback	Facilitate regulatory feedback loops
Ethical and legal review	Ensure that AI aligns with ethical and legal standards	Protected by ethical and legal safeguards	Uphold ethical standards in AI use	Ensure compliance with regulations	Adhere to ethical and legal standards	Lead ethical and legal reviews	Conduct legal reviews and compliance checks

This “layered, sequential, strategic integrative approach” enables continuous improvement of the entire process. An initial assessment uses paradigms such as the quiz, vignette, and knowledge survey and historical data comparison to evaluate AI’s knowledge base and practical accuracy, which are iteratively refined and optimized by applying the feedback loop paradigm using insights from real-time monitoring and expert consensus followed by algorithm transparency and audits to ensure that the system’s decisions are understandable and justifiable.

For AI-based systems targeting specific or novel medical fields, the rare or complex simulation and scenario testing should be integrated alongside challenging question paradigms to push the boundaries of AI’s capabilities and uncover areas for innovation. The feedback loop paradigm should be implemented so that AI systems are regularly updated based on new clinical evidence, shifts in expert consensus, and outcomes from real-time monitoring to ensure that AI remains aligned with current medical standards and practices through continuous evolution and adaptive learning.

This evolution is maintained transparently in terms of how feedback and new data influence AI algorithms, fostering trust among health care professionals and patients. Regular ethical and legal reviews should accompany these updates to address any emerging concerns.

Throughout the process, which is dynamic, adaptive, and iterative, a broad range of stakeholders—including patients, health care professionals, ethicists, and legal experts—should be engaged. This ensures that diverse perspectives are considered, particularly in applying paradigms such as expert consensus, ethical and legal review, and real-time monitoring. As previously mentioned, integrating these paradigms creates an ongoing process for evaluating and improving AI in health care, acknowledging the complexity of medical decision-making and the importance of maintaining ethical standards and ensuring that AI systems are not only accurate and effective but also trusted and ethical components of health care delivery.

## Toward a Model of “Clinically Explainable, Fair, and Responsible Clinician-, Expert-, and Patient-in-the-Loop Artificial Intelligence”

Clinical decision-making is a cornerstone of health care, demanding a blend of knowledge, intuition, and experience. It is a dynamic process in which clinicians sift through patient data, balancing the effectiveness and risks of treatments against patient preferences and ethical standards with the goal of optimal health outcomes achieved through informed, evidence-based choices that respect patient autonomy and confidentiality [54-56].

As previously mentioned, clinical decision-making is built on 4 pillars: scientific evidence, clinical judgment, ethical considerations, and patient involvement. The integration of generative AI into this realm presents exciting possibilities and challenges—on the one hand, AI’s capacity to analyze vast amounts of medical data can enhance diagnosis, tailor treatments, and predict disease progression. However, its incorporation demands rigorous verification to align AI-generated insights with medical standards and ethical practices.

In this conceptual paper, to ensure the reliability of AI in clinical decision-making, various verification paradigms have been proposed. The quiz, vignette, and knowledge survey paradigm assesses AI’s proficiency in medical domains by using realistic scenarios to test its knowledge and contextual application incorporating frequentist and Bayesian reasoning in clinical diagnosis, whereas the historical data comparison paradigm examines AI recommendations against known clinical outcomes, assessing real-world accuracy. The expert consensus paradigm involves a panel of medical experts evaluating AI-generated diagnoses and treatment plans, whereas the cross-discipline validation paradigm cross-checks AI insights with those of professionals from different medical fields, ensuring comprehensive evaluation. In addition, the rare or complex simulation and scenario testing paradigm tests AI against a range of clinical scenarios, revealing its strengths and limitations. The false myth paradigm tests the AI’s ability to reject outdated concepts or information and content not substantiated by scientific evidence, whereas the challenging question paradigm assesses how AI handles nuanced medical issues. The real-time monitoring paradigm involves implementing AI recommendations in controlled environments to monitor patient outcomes. The algorithm transparency and audit paradigm focuses on understanding how AI reaches its conclusions, essential for clinician trust. The feedback loop paradigm ensures AI’s continuous improvement based on practical application feedback. Finally, the ethical and legal review paradigm ensures that AI recommendations comply with ethical guidelines and legal requirements. Each paradigm offers a unique perspective for verifying AI in clinical decision-making, and when used in combination, they provide a comprehensive framework for ensuring the accuracy and reliability of AI, crucial for its effective integration into health care. This blend of AI and traditional clinical expertise promises a future of enhanced health care delivery, marked by precision, efficacy, and patient-centered care.

The convergence of generative AI in clinical decision-making, when rigorously verified and integrated with traditional health care practices, paves the way for a model of “clinically explainable, fair, and responsible clinician-, expert-, and patient-in-the-loop artificial intelligence.” This model emphasizes not just the technical prowess of AI but also its comprehensibility, collaborative nature, and ethical grounding, ensuring that AI acts as an augmentative tool rather than an opaque, autonomous decision maker (“AI as a black box”). Clinically explainable AI demystifies the often complex and opaque decision-making processes of AI systems. In particular, the algorithm transparency and audit paradigm plays a crucial role here, ensuring that AI’s reasoning is accessible and understandable to clinicians. This transparency is vital for trust and effective collaboration between human experts and AI-based systems—clinicians need to understand the rationale behind AI-generated recommendations to make informed decisions, particularly in complex or critical cases.

This understanding would also facilitate discussions and interactions with patients, who are increasingly seeking active roles in their health care decisions. By demystifying AI outputs, health care providers can offer clear, comprehensible explanations to patients, fostering trust and informed consent. Incorporating clinicians and experts in the loop is, indeed, fundamental in realizing this model—the expert consensus and cross-discipline validation paradigms highlight the importance of human expertise in evaluating and interpreting AI-generated insights, with clinicians bringing invaluable context, experience, and judgment to the table, which are crucial for nuanced decision-making. AI in this context is a tool that augments but does not replace the clinician’s judgment. This collaboration ensures that AI recommendations are not only based on data and algorithms but also tempered by human insight and ethical considerations. Patient involvement is another cornerstone of this model—patient-centric care is increasingly recognized as a key component of quality health care.

The integration of AI in clinical decision-making should not diminish the patient’s role but, rather, enhance it. By providing tailored and precise medical insights, AI can empower patients with information that is specific to their condition and treatment options. This approach aligns with the growing trend toward personalized or individualized medicine, where treatments are tailored to individual patient profiles. AI can facilitate this by analyzing patient data in depth, offering insights that help with crafting personalized treatment plans. Moreover, engaging patients in the decision-making process aided by AI’s insights respects their autonomy and preferences, leading to better satisfaction and adherence to treatment plans. Implementing a clinically explainable, fair, and responsible clinician-, expert-, and patient-in-the-loop AI model also necessitates continuous learning and adaptation—the feedback loop paradigm ensures that AI systems evolve based on real-world outcomes and clinician inputs. This ongoing refinement is crucial for the AI-based tool to stay relevant and effective in the ever-changing landscape of medical knowledge and practice.

Finally, the ethical and legal review paradigm ensures that AI recommendations are continually assessed for ethical and legal compliance, an aspect critical in maintaining public trust and upholding professional standards. Trust in this context extends beyond mere reliability to include ethically relevant and value-laden aspects of AI systems’ design and use. This broadened understanding of trust aims to encompass concerns about fairness, transparency, privacy, and the prevention of harm, among others. While pure epistemic accounts of trust focus solely on rational and performance-based criteria, more broadly speaking, trust encompasses the full spectrum of ethical considerations necessary for truly trustworthy AI, fully integrating ethical considerations into the core of what it means for an AI system to be considered trustworthy. AI-based systems not only function effectively and reliably but also and especially operate within ethical boundaries, adhering to ethical standards and principles that respect human autonomy, prevent harm, and promote fairness and transparency [57].

In summary, the envisioned model of AI in health care is one in which AI acts as an intelligent, transparent, and adaptable assistant in the complex process of clinical decision-making, enhancing rather than replacing human expertise and keeping clinicians, experts, and patients central to the decision-making process. This approach not only leverages the strengths of AI in data processing and pattern recognition but also upholds the irreplaceable value of human judgment, experience, and ethical reasoning, all crucial for delivering high-quality patient-centered health care.

## Current State of the Art and Future Directions

Currently, in a great portion of articles, the authors have limited themselves to querying the AI-based tools on a variety of topics without fully leveraging their potential. While that was understandable at the beginning of the revolution posed by LLMs, when early fascination and curiosity were prevalent, it is time to go beyond just chatting with ChatGPT and shift toward a deeper, comprehensive, and robust assessment of the capabilities of smart chatbots in real-world clinical settings. Researchers should make responsible use of AI; use standardized reporting guidelines [58]; systematically compare different types of AI-based tools; evaluate the accuracy, repeatability, and reproducibility of the tools; and incorporate ethical and legal considerations. Validated and reliable reporting checklists are essential for ensuring that research findings and advancements are communicated clearly and consistently, facilitating comparative analyses across different AI-enhanced tools. This will help not only in identifying the most effective solutions but also in uncovering potential biases, limitations, and areas for improvement. By systematically comparing different AI-based tools and rigorously evaluating their performance, the research community can establish a benchmark for what constitutes successful integration of AI in clinical settings. A composite set of performance and outcome metrics is essential for validating the reliability of AI in clinical applications and for ensuring that tools can be confidently used across various settings without loss of performance quality. Currently, only accuracy is being investigated, with only a few studies exploring the repeatability and reproducibility of AI-generated medical responses and recommendations.

Scholars can harness the 11 paradigms proposed in this paper to make AI-enhanced applications more clinically relevant and meaningful as well as robust and safe.

## Conclusions

Generative AI holds immense promise in enhancing clinical decision-making and offering personalized, accurate, and efficient health care solutions. However, ensuring that this technology produces evidence-based, reliable, impactful knowledge is paramount. By using paradigms and approaches such as those outlined in this conceptual paper, the medical and patient communities can better leverage the potential of AI while safeguarding against misinformation and maintaining high standards of patient care.

## Abbreviations

AI: artificial intelligence
ESHRE: European Society of Human Reproduction and Embryology
LLM: large language model
RCT: randomized controlled trial
